# A Mechanistic DNA Repair and Survival Model (Medras): Applications to Intrinsic Radiosensitivity, Relative Biological Effectiveness and Dose-Rate

**DOI:** 10.3389/fonc.2021.689112

**Published:** 2021-06-29

**Authors:** Stephen Joseph McMahon, Kevin M. Prise

**Affiliations:** Patrick G Johnston Centre for Cancer Research, Queen’s University Belfast, Belfast, United Kingdom

**Keywords:** radiation biology, computational biology, radiosensitivity, radiotherapy, Medras

## Abstract

Variations in the intrinsic radiosensitivity of different cells to ionizing radiation is now widely believed to be a significant driver in differences in response to radiotherapy. While the mechanisms of radiosensitivity have been extensively studied in the laboratory, there are a lack of models which integrate this knowledge into a predictive framework. This paper presents an overview of the Medras model, which has been developed to provide a mechanistic framework in which different radiation responses can be modelled and individual responses predicted. This model simulates the repair of radiation-induced DNA damage, incorporating the overall kinetics of repair and its fidelity, to predict a range of biological endpoints including residual DNA damage, mutation, chromosome aberration, and cell death. Validation of this model against a range of exposure types is presented, including considerations of varying radiation qualities and dose-rates. This approach has the potential to inform new tools to deliver mechanistic predictions of radiation sensitivity, and support future developments in treatment personalization.

## Introduction

Radiotherapy remains a key modality in the treatment of cancer, a role which has expanded through the development of novel technologies enabling improved imaging of tumor targets and precise delivery of individually-tailored treatment plans (1). This physical precision has led to reduced doses to organs at risk, and improved treatment outcomes across a range of cancers.

However, in contrast to this physical precision, biological precision remains an under-explored avenue of treatment optimization. The majority of cancers are treated in a one-size-fits-all approach, with all patients with a given type of cancer receiving the same treatment dose and fractionation. While this has been successful at delivering effective treatments on the population level, there is now significant evidence of inter-patient heterogeneity in radiosensitivity which could be exploited to maximize patient benefit (2–4).

Efforts to reach this goal have been hampered by the difficulties in generating a robust model of how cells respond to ionizing radiation. While simple approaches such as the Linear Quadratic (LQ) model have proven effective at describing overall patient responses (5), the development of more detailed mechanistic approaches have proven challenging (6). Much of the mechanistic modelling of radiation responses has focused on the earliest stages of radiation interaction with biological systems. Here, it is known that differences in how densely energy is deposited within the cells (characterized in terms of Linear Energy Transfer, LET) impacts on the sensitivity of cells to a given dose of radiation, and numerous models seek to link this with the Relative Biological Effectiveness (RBE) of different types of radiation. A range of models have been developed and applied to predict physical differences in DNA DSB yield and distribution, using different underlying approaches and assumptions (7–15).

These physical differences in DNA damage represent only the first stage in radiation’s biological effects, however. These initial damages are then processed by a range of cellular repair processes, and the cell’s ability to detect, repair and respond to this data is critical in determining its radiation sensitivity, more so than the better-studied physical effects. In many models these biological effects are reflected through cell-specific fitting parameters which, while useful in describing individual systems, are of limited use in more general predictions or possible treatment personalization approaches. The most widely used of these include the Local Effect Model (LEM) (16) and the Microdosimetric Kinetic Model (MKM) (17), which have seen clinical adoption as tools to predict RBE in clinical carbon ion therapy for cancer. However, these approaches still lack patient-specific predictive power.

One approach which has seen significant attention in recent years is through the definition of genetic or transcriptional signatures of radiation sensitivity which can be used to personalize radiation therapy. While a number of signatures have been proposed (most notably the Radiosensitivity Index/Genetically Adjusted Radiation Dose approach, but also a range of others (18–21)) and some have been tested in limited clinical datasets, these signatures have proven highly heterogeneous, and often difficult to reproduce in independent studies or using other techniques, suggesting they are not capturing the true underlying mechanisms of radiation response (21).

A range of mechanistic modelling work has been carried out in this area, seeking to develop new approaches to link from early DNA damage to biological effects (22–26). However, in many cases these models are closely linked to original datasets, and there remains few models which have been independently validated across a wide range of cell types and endpoints, suggesting significant further development is needed in this area.

In this manuscript, we present a significant update to the Medras mechanistic model of DNA repair and cell death (27, 28). This model begins from initial distributions of DNA damage, and simulates how these DSBs interact to either repair successfully or misrepair and lead to significant genetic alterations, and the subsequent likelihood of cell death following these events. This model has been updated to enable the simulation of a range of radiation deliveries, including different dose-rates and fractionation schedules, and is validated against a broad panel of experimental endpoints for a range of radiation qualities. Significantly, this model makes use of no empirical cell-specific fitting parameters, potentially opening the way for its use as a platform for treatment personalization. This model is also available as an open-source tool for other investigators to explore and expand in their own work.

## Methods

**Figure 1:**
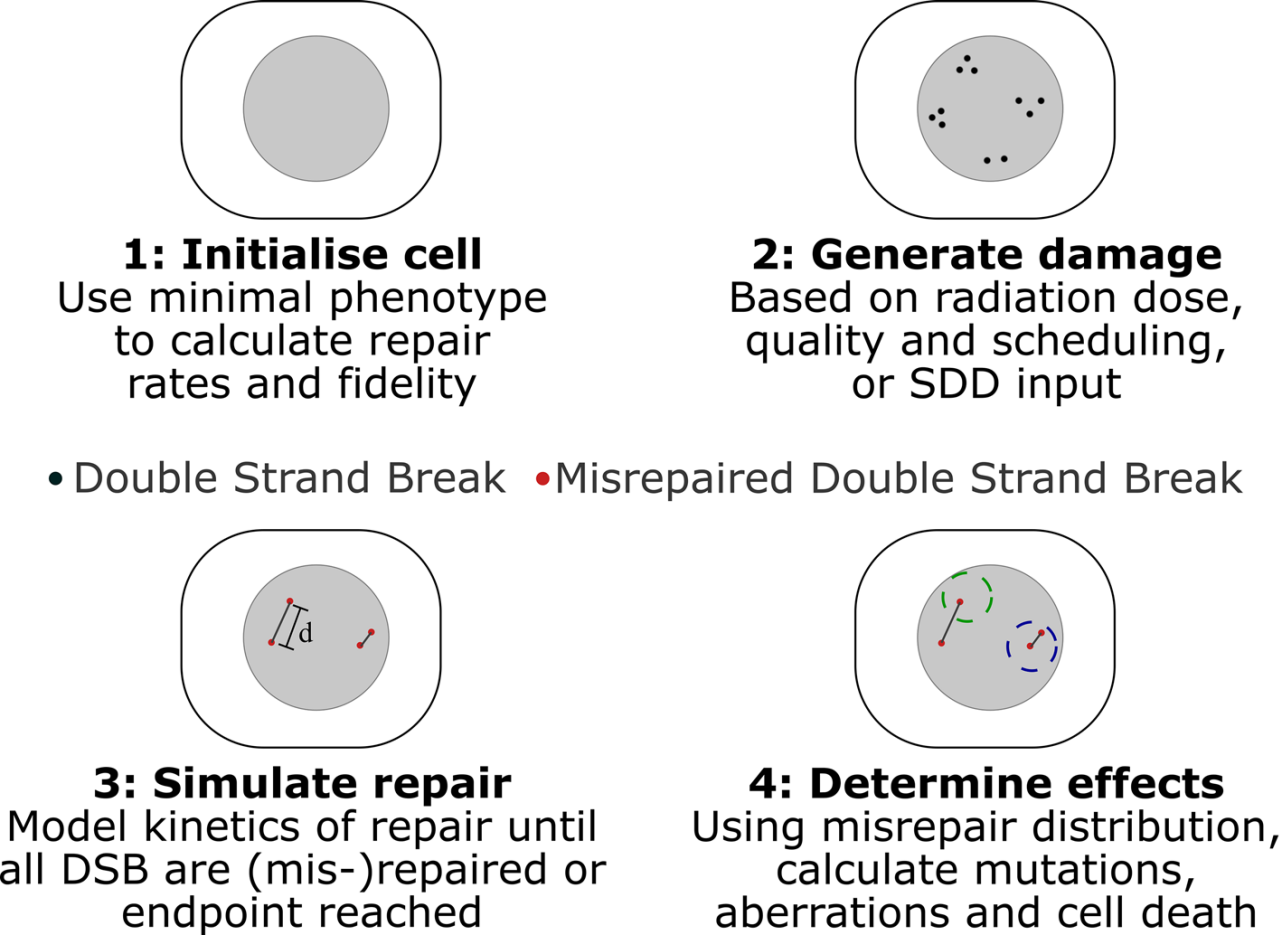
Schematic of key stages in Medras simulation of damage. 1) Cells are initialized, based on provided characteristics, determining rates and fidelity of repair. 2) Damage patterns are generated, either in Medras based on a described exposure or a provided SDD input file, incorporating details of track structure as relevant. 3) Repair is simulated as a function of time, tracking both amount of repair and distribution of misrepaired DSBs, with misrepair probability governed by inter-break separation d. Interacting breaks indicated by grey lines. 4) Biological endpoints are quantified. This can include number of unrepaired and misrepaired DSBs, but also mutations and chromosome aberrations, taking into account a simplified chromosome model, where spherical territories (dashed circles) are modelled to identify inter-chromosome (left) and intra-chromosome (right) aberrations.

Medras simulates the response of a cell to radiation beginning from a distribution of DNA Double Strand Breaks (DSBs), and simulates how these breaks may be (mis-)repaired as a function of time. Based on this simulated misrepair pattern, the probability of cell survival is then predicted, taking in various death pathways available to the cell in a particular condition. This is schematically illustrated in **Figure 1**, and each of the stages is summarized below.

### DNA Damage Distributions

Medras focuses on DNA Double Strand Breaks (DSBs) as the primary driver of radiation response, as there is strong evidence that they are the key lesion giving rise to cell death following radiation exposure (29, 30). It is thus assumed that the initial pattern of DSBs (in terms of both number and spatial distribution) determines the biological consequences of a given radiation exposure (31). Medras currently considers three key methods of simulating radiation exposures.

For the most commonly used sparsely ionizing radiation, such as X-rays and energetic electrons, a uniform distribution of damage is assumed, within a spherical nucleus. The number of breaks is taken to be directly proportional to the delivered dose of radiation, with a yield of DSBs of 5.738 GBP^-1^ Gy^-1^, corresponding to 35 DSB/Gy per human cell, in line with published studies (32). This assumption of a uniform distribution gives rise to a response which is purely determined by the dose delivered to the cell, as described in more detail below.

Two options are provided to describe the effects of particles with a higher Linear Energy Transfer (LET), such as protons or carbon ions. Firstly, Medras provides a tool to rapidly calculate distributions of DSBs around representative tracks for a range of particle LETs. To achieve this, radial track structures were modelled using Geant4 10.2 (33–35) and the Geant4-DNA toolkit (13, 36, 37). Ions of different species and energies were directed along the center of a cylindrical water phantom with radius 200 μm and depth 22 μm. Within the central 2 μm of depth, energy deposition from both the primary ion and secondary electrons were recorded and scored in terms of radial distance to the primary particle trajectory as it entered the scoring region. This provides a radial energy distribution, scored in logarithmic bins (smallest bin 0.1 nm radius, 100 bins per factor of 10 change in radius). Primary counts ranged from 600 to 20,000 depending on primary particle. Example radial energy distributions are presented in the **Supplementary Information**.

It is then assumed that all radiation types lead to the same number of DSBs per cell per unit dose - 5.738 GBP^-1^ Gy^-1^. The number of DSBs in a given radial bin around an ion track can thus be calculated as $\frac{E(r)}{E_{DSB}}$, where *E(r)* is the energy in the bin at a distance *r* from the ion track, and *E_DSB_* is the energy associated with the creation of on average one DSB. It should be emphasized that *E_DSB_* is independent of the distribution of the energy within the nucleus, so it is assumed that it leads to an average of one DSB whether it is uniformly distributed throughout the nucleus, or densely clustered around a single ion track.

This assumption enables the yield of DSB to be readily calculated for any given energy deposit. However, it also represents a degree of simplification, as there is some evidence that the yield of DSBs is affected by LET. However, robust quantification of an RBE for DSBs has proven challenging. Different DSB assays produce very different measurements for this value, with some identifying increases, some decreases, and some more complex patterns (38, 39). While the evidence as a whole suggests that an excess of DSBs is produced within the track of charged particles, the total size of this effect is small – with many assays suggesting at most an increase in DSBs of a factor of around 1.4 for particles with LETs of 100 keV/μm (39). By contrast, the RBE for cell killing at this LET is several-fold higher, suggesting the increased lethality per DSB, rather than an increase in the number of DSBs is the primary driver of increased RBE, similar to observations elsewhere in the literature (40).

As dose is defined as energy deposited per unit mass, *E_DSB_* is closely related to the volume of the nucleus. In particular, assuming a human cell which experiences 35 DSB/Gy, we can say $35=\frac{1\;Gy\times V_{Nuc}}{E_{DSB}}$, where *V_Nuc_* is the volume of the nucleus and a density of 1 g/cc has been assumed. If *E_DSB_* is expressed in keV and *V_Nuc_* is μm^3^, this can then be expressed as *V_Nuc_* =*5.16E_DSB_*, or equivalently $r_{nuc}=1.1\sqrt[{3}]{E_{DSB}}$, which provides a useful benchmark for the value of *E_DSB_*. *E_DSB_* is not determined *a priori*, and has instead been fit to observed RBE data, as described in previous publications (28) and summarized below. In this approach, a best-fitting value of *E_DSB_*=56.5keV has been obtained, equivalent to *r_nuc_*=4.23 *µm*, in agreement with typical estimates of cell nucleus radii.

Finally, this radial DSB distribution can be used to calculate the average number of intra-track DSBs as a function of distance from an average break within the track. The interaction rate of breaks within the track can then be calculated as described below, and combined with the inter-track break distribution (which is taken as random and uniform on average) to provide an estimate of the total rate of misrepair.

For very high-LET exposures, it is also important to note that each track will likely cause multiple DSBs and each exposure may only consist of a few particle traversals. This gives rise to a non-Poisson distribution of initial damage, and can significantly increase observed survival. To account for this, when the expected number of DSBs per track is greater than 0.5, cell responses as described below are simulated for a range of different incident particles, weighted assuming a Poisson distribution of tracks with a mean equal to that which delivers the prescribed dose, and the average responses are returned.

As an alternative approach to these averaged estimates of DSB induction for ions, damage distributions can also be imported using the Standard for DNA Damage (SDD) file format (41). The SDD format provides a standardized method for recording DSB damage from physical simulations so they can be imported into repair code, such as their spatial and temporal distribution, as well as genetic and break complexity info. Medras provides an interface through which these files can be read and repair simulated within them, and can also export representative damage distributions based on the assumptions above for reference. This facility for arbitrary input enables the simulation of full details of DSB distributions without any simplifying assumptions, and the possibility of benchmarking repair predictions comparing different DNA damage models.

Regardless of the method used to generate these DSB distributions, they can then be imported into the core Medras repair simulation, and used to predict radiation responses as described below.

### Repair Rates

Within Medras, breaks are separated into broad categories of ‘simple’ and ‘complex’. Currently, the model deliberately does not explicitly consider the details of break complexity on the level of features such individual base or strand damages, local chromatin environment, or other biological factors, as it remains unclear which of these features are key to determining break repair process (42). Instead, within Medras break complexity is assigned randomly with a probability *p_complex_* at break creation, or it can be read from data provided in the SDD file.

Double strand breaks can be repaired by one of three pathways – Nonhomologous End Joining (NHEJ), Homologous Recombination (HR) and Microhomology Mediated End Joining (MMEJ, also known as alternative end joining, alt-EJ), depending on cell cycle phase and pathway activity (43–45). In normal cells, simple breaks are repaired by NHEJ in all cell cycle phases, while complex breaks are repaired by NHEJ in G1, and HR in later phases once replicated sister chromatids are available to act as a template. However, in cells with repair defects, some DSBs which attempt to repair through these pathways will fail and instead be repaired by the backup MMEJ pathway, with probability *p_fail_*.

This gives rise to up to three populations of breaks, repaired by different kinetics. “Fast” repair represents the simple breaks which are repaired by NHEJ throughout the cell cycle. “Slow” repair represents complex breaks which require more time to be processed, either by NHEJ following a degree of end processing to reduce end complexity (in G1) or HR (in S and G2) (46). Finally, a subset with “Very Slow” repair kinetics is present in cells with DNA repair defects requiring the use of MMEJ, which is significantly slower than any other process.

**Figure 2:**
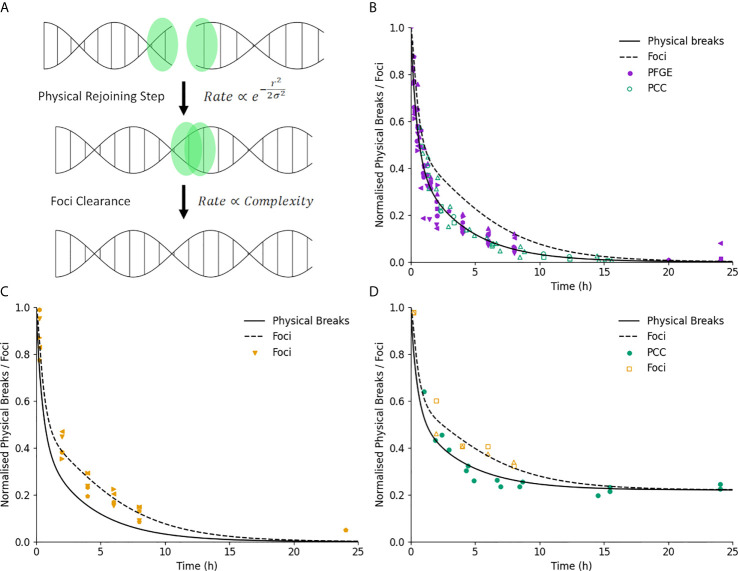
Illustration of DNA repair kinetics. **(A)** Key modelled stages in DNA repair. Break ends are initially free, and interact physically with a nearby end with a rate which is related to the break complexity and initial separation. Once joined, the associated foci is cleared after a delay which depends only on break complexity. **(B)** Break kinetics for physical breaks and foci (solid and dashed line) in normal cells. Points represent measured breaks *via* PFGE (solid) and PCC (open). Error bars not shown for clarity. **(C)** Break kinetics compared to measurements *via* foci (points) illustrating impact of foci clearance on repair kinetics. **(D)** Measurements of repair in ATM-defective lines for both physical breaks (filled) and foci (open), showing similar impact on kinetics and final breaks for both endpoints.

Medras simulates all of these repair pathways as a two-step process, schematically illustrated in **Figure 2A**. Each DSB initially consists of two free ends, which are rapidly bound by a selection of sensing and repair proteins. Such breaks are detected on both ‘physical’ assays which detect break structure such as Pulse Field Gel Electrophoresis (PFGE) or Premature Chromosome Condensation (PCC), as well as through immunofluorescent staining of associated repair proteins. Pairs of break ends can then be bound together to restore the physical structure of DNA. At this stage, the DSB ends are no longer free, can no longer interact with other break ends, and the break will not be detected through physical assays. However, repair proteins remain bound at the site of the break for some time after this physical rejoining, and it is not until these proteins have been cleared that the break will no longer be detected by immunofluorescence.

Both of these stages in repair are simulated in the analytic model as simple exponential processes. For a simple acute exposure which induces *N_0_* initial DSBs, the kinetics of physical breaks *N_phys_* is given by:

(1) $$N_{phys}=N_{0}\left(p_{f}e^{-\lambda_{f}t}+p_{s}e^{-\lambda_{s}t}+p_{m}e^{-\lambda_{m}t}\right)$$

Where *p_x_* and *Λ_x_* are the probability of a break being repaired by pathway *x* and the associated repair rate, where *x* corresponds to is fast (*f*), slow (*s*) or MMEJ (*m*) repair. In repair competent cells, these probabilities are given by *p_f_*=(1-*p_complex_*), *p_s_=p_complex_* and *p_m_*=0 If either one or both of the preferred repair mechanism are knocked out, then these probabilities are updated to reflect the rate of failure. For example, if a cell in G2 is deficient in HR, the repair probabilities would become *p_f_* = *(1-p_complex_)*, *p_s_=p_complex_(1-p_fail_)*, and *p_m_=p_complex_p_fail_*. A full tabulation of possible combinations of break complexity and repair capacity and the resulting repair rates is presented in the **Supplementary Information**. Each of the rate repair coefficients is taken as a model fitting parameter.

In a more general case where breaks are not initially generated in a single acute exposure but rather over some time, the number of breaks repaired by each pathway can be described by a rate equation. For example, for breaks repaired through the fast pathway the rate of change in physical breaks repaired with fast kinetics, $N_{phys}^{f}$, is:

(2) $$\frac{dN_{phys}^{f}}{dt}=-\lambda_{f}N_{phys}^{f}+p_{f}k\dot{D}(t)$$

Where $\dot{D}(t)$ is the dose rate at time *t*, and *k* is the yield of double strand breaks per Gy. The first term thus represents the standard exponential decay of breaks, and the second term represents the number of fast-repairing breaks induced as a function of time. Similar expressions can be formulated for breaks repaired through the other pathways. This can be readily solved numerically to provide the kinetics of physical DSBs for an arbitrary pattern of dose delivery. In particular, time-varying dose rates can be considered by using a time-dependent value of $\dot{D}(t)$, which can include fractionation by modelling inter-fraction gaps as a period where $\dot{D}(t)=0$. At present this expression is only accurate for quiescent cells, as proliferation during exposures is not incorporated.

For joined breaks which still bear repair proteins, the expression is somewhat more complex, as these are not created directly by radiation, but rather after some delay associated with initial end joining. We can define the kinetics of the number of protein-bearing rejoined breaks being repaired with fast kinetics, $N_{prot}^{f}$ as:

(3) $$\frac{dN_{prot}^{f}}{dt}=\lambda_{f}N_{phys}^{f}-\nu_{f}N_{prot}^{f}$$

Where *v_f_* is the rate at which proteins are cleared in this pathway. Similar expressions apply to the other pathways. In general, this then introduces a complex dependence on the pattern of dose-rate and physical break repair, and requires numerical solutions for many approaches. For the specific case of a single acute exposure inducing *N_0_* initial breaks, however, this can be explicitly solved to give (see **Supplementary Information**):

(4) $$N_{prot}^{f}=\frac{N_{0}p_{f}\lambda_{f}\left(e^{-\lambda_{f}t}-e^{-\nu_{f}t}\right)}{\nu_{f}-\lambda_{f}}$$

Which gives rise to an initial rise and then fall in the number of protein-bearing joined breaks, as expected. Of more relevance to experimental endpoints, immunofluorescence studies of markers such as γH2AX foci thus measure the total number of both physical and protein-bearing breaks, for a total count of visible foci being repaired with fast kinetics,$N_{foci}^{f}$, of:

(5) $$N_{foci}^{f}=N_{phys}^{f}+N_{prot}^{f}=N_{0}p_{f}e^{-\lambda_{f}t}+\frac{N_{0}p_{f}\lambda_{f}\left(e^{-\lambda_{f}t}-e^{-\nu_{f}t}\right)}{\nu_{f}-\lambda_{f}}=N_{0}p_{f}\frac{\nu_{f}e^{-\lambda_{f}t}+\lambda_{f}e^{-\nu_{f}t}}{\nu_{f}-\lambda_{f}}$$

And similar expressions for each of the other pathways. Using these expressions, the yields of both physical breaks and foci can be calculated for any acute or protracted radiation exposure. These can both be used for direct comparison with experimental observations of DNA repair kinetics, as well as to support calculations of misrepair following different radiation exposures.

### Repair Fidelity – Analytic

As breaks repair, the model then simulates the probability that each break undergoes either ‘correct’ repair or misrepair. Misrepair is here defined as when ends from two distinct DSBs are joined together, leading to at least some degree of genetic rearrangements and potentially significant genetic alterations. In Medras, we define the probability of any given pair of ends being joined together as

(6) $$\zeta (r)\propto e^{\frac{-r^{2}}{2\sigma^{2}}}$$

Where ζ(*r*) is the relative interaction rate of two breaks separated by a distance *r*, and *σ* is a scaling coefficient related to the characteristic rejoining range of breaks within the cell. As the two ends of a single DSB are naturally in close proximity, for correct repair ζ ≈ 1, while the rate of incorrect joining depends on the number and distribution of other breaks within the nucleus.

The total rate of misrepair depends on the sum of these rates of incorrect misrepair, that is

(7) $$\eta_{i}=2\sum_{j\neq i}^{N}\zeta \left(r_{i,j}\right)$$

Where the total misrepair rate for the *i*-th break, *n_i_* is equal to the sum of (*r_i,j_*) over the other *N-1* breaks, multiplied by two to reflect each break consists of two free ends. For a single break repair event, the probability of correct repair is then given by the rate of correct pairing as a fraction of the total rate, that is:

(8) $$p_{correct}=\frac{1}{1+\eta_{i}}$$

Where we assume the rate of correct interaction is equal to 1. We have previously shown (27) that for a situation where all breaks are fully repaired, the total probability of each break being correctly repaired is given by:

(9) $$P_{correct}=\frac{1-e^{-\eta }}{\eta }$$

Which was validated against a range of experimental and theoretical benchmarks in previous work (27, 28). However, this formulation is only applicable for complete repair from a single fraction – it cannot be applied to scenarios of e.g. fractionated or prolonged exposures. A general summation of equation 8 reflecting the discrete nature of breaks is not possible, but it can be closely approximated by a continuous integration for more than a few breaks. However, this cannot be simply used directly, as when a misrepair event happens, the other ends of the two involved DSBs have lost their partner, and thus are no longer able to correctly repair. This necessarily leads to an additional misrepair events following a first event, which leads to a small but significant increase in misrepair events after a first repair event. To take this into account, we add an additional term of $n_{i}^{2}$ to the misrepair rate, reflecting the first-order contribution of misrepaired breaks. Thus we have:

(10) $$P_{correct}=\int^{}p_{correct}(\eta )d\eta =\int^{}\frac{1}{1+\eta_{i}+\eta_{i}^{2}}d\eta$$

To solve this, we define *η* = *η'N*, where *N* is the number of breaks present and *η'* is the average value of ζ across all breaks within the system. We can thus say the number of correct repair events when the number of breaks repaired goes from *N_0_* initial breaks to *N_1_* final breaks is:

(11) $$N_{correct}=\int_{N_{1}}^{N_{0}}\frac{1}{1+\eta^{\prime }N+{\eta^{\prime }}^{2}N^{2}}dN={\left[2atan\left(\frac{2\eta^{\prime }N+1}{\sqrt{3}}\right)\right]}_{N_{1}}^{N_{0}}$$

Substituting in *N*_0_ and *N*_1_ into the final part of equation 11, simplifying through trigonometric identities and dividing by *η'*(*N*_0_ – *N*_1_) to express this as a probability gives a probability of correct repair of:

(12) $$P_{correct}=\frac{2}{\sqrt{3}\;\eta^{\prime }\left(N_{0}-N_{1}\right)}atan\left(\frac{\sqrt{3}\;\eta^{\prime }\left(N_{0}-N_{1}\right)}{2+\eta^{\prime }\left(2\eta^{\prime }N_{0}N_{1}+N_{0}+N_{1}\right)}\right)$$

While somewhat unwieldy, this gives a flexible way to predict the degree of correct and incorrect repair following any amount of repair, for any initial and final yield of DSBs. This enables generalized predictions to be made for any combination of fractionated exposures, or prolonged exposures through numerical integration. In the special case of complete repair *(N_1_=*0*)*, *P_correct_* simplifies to

(13) $$P_{correct}=\frac{2}{\sqrt{3}\;\eta^{\prime }N_{0}}atan\left(\frac{\sqrt{3}\;\eta^{\prime }N_{0}}{2+\eta^{\prime }N_{0}}\right)$$

Which can be compared to the form in equation 9 to confirm it accurately reproduces misrepair rates at a broad range of doses (see **Supplementary Information**). This enables the analysis of a broad range of scenarios not covered by the original Medras model for further validation and testing. by the original Medras model, and the integration of new endpoints for validation and ras model, and the integration of new sce

The value of *η'* can be estimated in a number of ways. For a known break distribution it can be calculated explicitly, while for a uniform break distribution within a spherical nucleus it can be estimated analytically. As described previously, this analytical estimate is given by:

(14) $$\eta^{\prime }(R,\sigma )=\frac{6}{4\pi R^{3}}\theta (R,\sigma )$$

Where *R* is the radius of the nucleus and *θ* is the rejoining rate between two randomly placed DSB ends, given by:

(15) $$\theta (R,\sigma )=\frac{2\pi \sigma^{2}}{R^{3}}\left(\sqrt{2\pi }\;R^{2}\sigma \;erf\left(\frac{R\sqrt{2}}{\sigma }\right)-e^{\frac{-4R^{2}}{2\sigma^{2}}}\left(\sigma^{2}-R^{2}\sigma^{2}\right)+\left(\sigma^{2}-3R^{2}\sigma^{2}\right)\;\right)$$

To incorporate intra-track events, based on the break separation distributions as described above we can calculate an *η_track_* value, reflecting the average intra-track contribution for a randomly given break for a given particle and energy. This can then be combined with the *η*′ value described above to give the total misrepair rate per track, that is *η*′*_track_*=*η*′+*η_track_*, and this can be used directly in equation 12 or 13 to calculate the rates of correct repair, incorporating intra-track effects in an analytic way.

Finally, even in the absence of incorrect end joining, repair pathways have an inherent probability of misrepair. For NHEJ and MMEJ, this is reflected with an additional reduction in the total rate of correct repair independent of binary misrepair, defined as $P_{correct}=\mu_{x}\left(\frac{2}{\sqrt{3}\;\eta^{\prime }N_{0}}atan\left(\frac{\sqrt{3}\;\eta^{\prime }N_{0}}{2+\eta^{\prime }N_{0}}\right)\right)$, where *μ_x_* is a process-specific fidelity factor. For HR, it is instead assumed that repair is always correct, giving *P_correct_*=1.

### Repair Fidelity – Monte Carlo

As an alternative to the above analytic approach, physical misrepair rates can also be simulated *via* Monte Carlo approaches. This uses a simple sampling approach to replicate the assumptions of the analytic model, but enables a flexible calculation for more complex DSB distributions (as those imported from external packages using the SDD interface (41)), and enables the temporal impact of misrepair to be accounted for.

The Monte Carlo simulation begins from a full distribution of all of the DSBs resulting from each exposure. It calculates and stores the full set of ζ*_i,j_* interaction rates for every pair of break ends, and then calculates the total interaction rate *η_i_* for each break end with all other free break ends, including the correct partner. This total interaction rate then scales the effective repair rate for a given break, *Λ_i_*, as:

(16) $$\lambda_{i}=\lambda_{x}\eta_{i}$$

Where *Λ_x_* is the repair rate associated with the pathway through which the break end will be repaired as described above (*f,s,m*), and *Λ_i_* is the effective rate of repair for the *i*-th break. This enables the Monte Carlo model to reflect the slight elevation in repair rate seen in regions with many DSBs, and the significant fall in repair rate if the correct partner end is repaired, which substantially reduces *η_i_*.

For each break end, the associated time of repair is then randomly sampled as:

(17) $$t_{i}=-\frac{\log(X)}{\lambda_{i}}$$

Where *t_i_* is the time until the break end is repaired, and *X* is a randomly uniformly distributed value between 0 and 1, replicating the exponential distribution of repairs. This approach is conceptually similar to those used in, for example, independent reaction times modelling in chemistry simulations (47).

The simulation then proceeds by identifying the break end with the smallest *t_i_* to be the next end to undergo repair. A partner end is then selected at random from all remaining ends, with probabilities weighted by their interaction rates ζ*_i,j_*. This pair of break ends are then logged as a repair event, removed from the simulation, and values for *η_i_* and *t_i_* are updated for all remaining breaks. Then the process is repeated with the next-smallest *t_i_* until all break ends have been rejoined.

Protracted exposures are modelled in a similar fashion. In addition to ‘active’ breaks which have already been created, a list of breaks induced at later timepoints is also stored. If the next repair is predicted to occur after a new break will be induced, that break will instead be added to the simulation, and *η_i_* and *t_i_* updated for all breaks as above to reflect the newly available repair partners.

Once all breaks have been repaired, a full list of repair events is then available, and can be used to plot the kinetics of repair of physical breaks, have a delay associated with repair protein clearance added to predict the yields of foci as for analytic breaks, or the patterns of misrepair can be analyzed to produce predictions of not only total misrepair, but also model-specific information on consequences of misrepair such as affected genes, chromosomes, and types of aberrations form, if underlying data is available.

This approach has been shown to accurately reproduce the behaviors of the analytic approach, as illustrated in the **Supplementary Information**.

### Misrepair Consequences

Misrepair events represent a broad category of events, ranging from small deletions to large-scale chromosomal rearrangements. In addition to simply predicting the yield of misrepairs, Medras also estimates the yield of several relevant types of alteration, particularly mutations and significant chromosomal aberrations. These have been described in previous work (27), and the concepts are summarized here for completeness.

Chromosome aberrations are the most significant class of genetic rearrangement for cell survival, potentially leading to large genetic losses or aberrant chromosomes which cannot separate during mitosis. They reflect large-scale rearrangements of chromosome structure, and can be classified as *inter-* or *intra-* chromosome, depending on which chromosomes contained the DSB ends involved in the repair. As a simplified analytic model of chromosome structure, chromosomes are modelled as spheres packed within the nucleus with radius $r_{c}=\frac{R}{\sqrt[{3}]{n_{c}}}$, where *n_c_* is the number of chromosomes in the nucleus. While this neglects variations in factors such as chromosome size and packing, as it focuses on average rates across the whole nucleus the impact of these factors is reduced.

From this, when misrepair occurs the probability of the interaction being intra-chromosome is given by the average interaction rate within a chromosome compared to that throughout the nucleus, that is:

(18) $$P_{intra}=\frac{\theta (r_{c},\sigma )}{\theta (R,\sigma )}$$

A second classification is whether the exchange is symmetric (both resulting chromosomes contain a centromere) or asymmetric (at least one acentric fragment is produced). Symmetric exchanges leave a relatively intact chromosome structure and are typically non-lethal, while asymmetric exchanges include acentric fragments, dicentrics, rings and other rearrangements which are often incompatible with cell survival (48). As the symmetry of the break is solely determined by the alignment of DSB ends which are otherwise treated as identical, this model assumes symmetric and asymmetric exchanges occur with equal frequency, *P_asym_*=0.5. Thus, the number of deletion (asymmetric intra-chromosome) and dicentric (asymmetric inter-chromosome) events can be calculated as *N_dic_*=0.5N*_mis_*(1-*P_intra_*) and *N_de_l*=0.5*N_mis_P_intra_*, where *N_mis_* is the number of misrepaired breaks as calculated above.

The size of deletions is also important for their lethality. By assuming that the separation of breaks in base pairs increases monotonically with distance between the break ends, the size of a deletion can be given by $D=\frac{2Lr_{D}^{3}}{R^{3}}$, where *L* is the total length of all chromosomes and *r_D_* is the separation of the break ends. The rate of deletions smaller than D is given by the rate of misrejoiing events over distances shorter than *r_D_* , given both events occur within the same chromosome. This is given by $P_{del<D}=\frac{\theta (r_{c},\sigma ,r_{D})}{\theta (r_{c},\sigma )}$ where the generalized *θ* is given by:

(19) $$\theta \left(r_{c},\;\sigma ,\;r_{D}\right)=\frac{\pi \sigma^{2}}{4r_{c}^{3}}\left(8\sqrt{2\pi }r_{c}^{3}\sigma \;erf\left(\frac{r_{d}\sqrt{2}}{\sigma }\right)-e^{\frac{r_{D}^{2}}{2\sigma^{2}}}\left(r_{D}^{4}+4r_{D}^{2}\left(\sigma^{2}-3r_{c}^{2}\right)+16r_{D}r_{c}^{3}+8\sigma^{2}\left(\sigma^{2}-3r_{c}^{2}\right)\right)+\left(8\sigma^{4}-24r_{c}^{2}\sigma^{2}\right)\right)$$

And we can then express the number of deletions larger than some threshold size as *N_del_>_D_*=0.5*N_mis_P_intra_*(1-*P_del_<_D_*). For this work, we define a ‘large deletion’ of the type typically associated with cell death as one of 3 MBP or greater size, as this has been shown to correlate well with cell death in Giemsa-stained cells (49).

This relationship between spatial separation and genetic separation can also be used to calculate the rate of inter-arm interactions (relevant for chromosome aberration visibility in G2) and the rate of mutation in a particular gene (by calculating the probability a misrepair event spans some or all of the gene of interest). For the specific case of mutation, mutations can also be caused even during correct end joining, where NHEJ can introduce small changes in sequence to one or a few base pairs, affecting the sequence but not overall structure. This is accounted for with a point mutation probability, *p_mut_*, which applies when a break is correctly repaired within a gene but may still cause a mutation.

### Cell Death

Medras considers three cell key mechanisms – genetic damage which renders the cell unviable, apoptosis, and mitotic catastrophe. The impact of genetic damage is determined directly from the yields of misrepair, and in particular lethal chromosome aberrations. We define lethal aberrations as those which prevent segregation at mitosis (dicentrics, rings) or those which remove enough genetic material to prevent cell function (large deletions). Cell death in quiescent cells has been shown to correlate extremely well with such aberrations measured in Giemsa-stained cells, an assay which is sensitive to deletions greater than 3 MBP in size (49). Thus, the rate of cell death from such aberrations in quiescent and G1 cells is given as $S=e^{-N_{dic}-N_{del>3MBP}}$, assuming aberrations occur with a Poisson distribution. For cells irradiated in G2 a single large deletion is insufficient to lead to cell death, as DNA has already been replicated and both daughter cells must see genetic loss to be rendered unviable. As a result, survival in such cells is given by *S=e^-Ndic-Ninterarm^*. This neglects the small contribution of cells dying due to multiple independent large deletions, but this is rare at relevant doses.

In addition to these misrepair-driven events, the presence of unrepaired breaks at mitosis can also lead to cell death through mitotic catastrophe. This may be due to either newly formed breaks, or escaping the G2 DNA damage checkpoint (observed when fewer than 20 DSBs remain (50)). Extensive experimental evidence (51) indicates that the dependence of mitotic catastrophe on induced DSBs is a simple exponential kinetic, with similar rates across cell lines. Medras thus models the probability of successfully completing mitosis as *S_mitosis_=e^-ϕNm^*, where *N_m_* is the number of DSBs present in mitosis, and *ϕ* is a rate constant shared across all cells.

Finally, cells can also undergo long-term arrest (senescence) or programmed cell death (apoptosis) following irradiation. These are complex processes depending on a range of genetic and environmental factors, but play a particularly important role in *in vitro* survival in G1, where they are most commonly observed. Experimental quantification of their relative importance remains difficult (52), and even a partial systems biological model remains outside the scope of this work. Instead, a simple empirical approach is applied, based on experimental evidence which shows that the likelihood of cells escaping the G1 checkpoint is an approximately exponential function of dose. Thus, as with mitotic catastrophe, the probability of escaping apoptosis in G1 is modelled as $S_{apop}=e^{-\psi_{x}N_{G1}}$, where *N_G1_* is the number of DSBs induced in G1. For cells irradiated while non-cycling or in other phases of the cell cycle, apoptosis does not occur.*Ψ_x_* has two possible values. For cells with fully functional DNA damage sensing and apoptotic processes, it has the value *Ψ_full_*. However, dysregulation or mutation of this pathway is very common in many cancer cells, particularly through mutation in TP53 and associated genes (53). As a result, this process is inhibited in many cells, and happens at a much lower rate of *Ψ_base_* The exact values of these two rate parameters was fit to experimental data in acutely irradiated cells as described below.

### Data Acquisition

To test and validate the model, a broad panel of data was acquired from the literature. As described in previous work (27, 28), data was obtained for DNA repair kinetics, misrepair *via* PFGE, chromosome aberrations, mutation rates, and cell survival following a range of exposure conditions. Values were extracted from published tables or figures along with uncertainties. An additional 5% uncertainty was added to all points to reflect uncertainties in data extraction.

For all experimental data used in this work, the cell line(s) used was identified, and related back to published datasets to determine a set of key cell-specific features. These are the genome size, chromosome number, NHEJ repair capacity, HR repair capacity, activity of G1 arrest (typically *via* p53 status) and the cell cycle phase of the irradiation. These parameters impact on response pathways as summarized in **Table 1**, and are the only cell-specific parameters used in a given simulation. No fitting parameters are adjusted on a cell- or experiment-specific basis.

**Table 1:** List of cell-specific features which define the minimal radiation phenotype used to predict the sensitivity of cells in this model.

Radiation Phenotype Parameters	
Parameter	Description
Genome size	Total genome size of cell in MBP
Chromosome number	Total number of chromosomes in cell
NHEJ repair capacity	Availability of NHEJ pathway
HR repair capacity	Availability of HR pathway
G1 Arrest function	Availability of G1/S phase damage arrest checkpoint
Cell cycle phase	Phase of cell during irradiation (specified as single phase or asynchronous)

All parameters determined from published literature and genetic status, without free fitting parameters.

A number of different data types were extracted from a range of publications to characterize different endpoints. For DNA repair kinetics, data was obtained for measurements of chromosome breaks measured using premature chromosome condensation (PCC) (54, 55), DSBs measured using Pulse Field Gel Electrophoresis (PFGE) (56–58), and DSB foci measured using immunofluorescent labelling (59, 60). Misrepair rates were obtained from PFGE measurements (61, 62). Mutation data was obtained for gross and point mutations in the HPRT gene (63–65). Yields of total chromosome aberrations measured through Giemsa staining were obtained for normal human cells (66–70), human-hamster hybrid cells (71), and NHEJ-defective cells (72) for acute exposures, and a number of human cells exposed at low dose rates (70, 73–75). Clonogenic survival data was obtained for a range of human (49, 55, 60) and hamster lines, including NHEJ-defective sublines (32, 76–78). Clonogenic survival values were also obtained for a number of cell lines exposed at varying dose rates to validate low dose-rate predictions (79–93).

To provide broader datasets for overall predictions of intrinsic sensitivity and to analyze the effects of RBE on survival, the proton RBE dataset published by Paganetti (94) was used for basic model fitting. This analysis focused on single-fraction exposures of adherent cells in oxic conditions, excluding exposures where the primary particle had an extremely low range (<1 cell diameter), or very limited dose rantes (max dose < 2 Gy). For each experiment which satisfied these conditions, the cell line, proton LET, and X-ray and proton α and β values were extracted. Mean Inactivation Doses (MID) were then calculated based on provided α and β parameters to characterize the overall sensitivity of the cells. The MID is defined as the average dose required to kill a cell in the population, equivalent to the area under the LQ response curve, and is given by

$$\int_{0}^{+\infty }e^{-\alpha D-\beta D^{2}}\;dD,$$

with units of Gy. This was used as a measure of overall survival, and to fit RBE parameters as described below.

To validate the RBE model predictions, the Particle Irradiation Data Ensemble (PIDE) (95) was used as a validation dataset, as it included proton data together with a range of other ions. For validation, all proton experiments not represented in the Paganetti dataset, as well as all carbon ion exposures were extracted from the PIDE, and analyzed in the same fashion as above to calculate MIDs and the resulting RBEs. This data was not used for fitting, but instead to test predictions made using parameters fit to the lower LET proton dataset.

### Model Parameter Fitting

The full set of model parameters used in this work is presented in **Table 2** together with their best-fitting values. To obtain these parameter values, the model was implemented in Python and fit using nonlinear regression in a number of stages, as described in previous work (27, 28). Some details on parameter covariance are presented in the **Supplementary Information**.

**Table 2:** Best-fit MEDRAS model parameters with uncertainty.

**DNA Repair Parameters**		
Parameter	Interpretation	Value
**σ**	Rejoining range	0.0418 ± 0.0003 R
**μ_NHEJ_**	NHEJ misrepair probability	0.985 ± 0.001
**μ_MMEJ_**	MMEJ misrepair probability	0.44 ± 0.05
**p_m_**	Point mutation probability	0.046 ± 0.004
**p_c_**	Complex break probability	0.43 ± 0.02
**p_fail_**	Repair failure probability	0.74 ± 0.09
**λ_f_**	Fast repair rate	2.1 ± 0.2 h^-1^
**λ_s_**	Slow repair rate	0.26 ± 0.02 h^-1^
**λ_m_**	MMEJ repair rate	0.0085 ± 0.001 h^-1^
**ν_f_**	Fast foci delay	8.1 ± 0.9 h^-1^
**ν_s_**	Slow foci delay	0.41 ± 0.09 h^-1^
**Survival model parameters**		
**Φ**	Mitotic catastrophe rate	0.014 ± 0.001 DSB^-1^
**ψ_full_**	Full apoptosis rate	0.012 ± 0.001 DSB^-1^
**ψ_base_**	Base apoptosis rate	0.0007 ± 0.0002 DSB^-1^
**High LET parameters**		
**E_DSB_**	Average energy per DSB	56.5 ± 15 keV

The first stage of the model focused on the DNA repair model. In this, a single simultaneous fit was performed across all DNA repair model parameters, fitting to data on repair kinetics, misrepair, mutation and aberration in a single step. A weighted least-squares regression was performed using Scipy (96) across all data in the dataset. Overall performance was good, with a mean *χ^2^* of in a single step. oss all DNA repair model parameters, fitting to data on repair kinetics, misrepair, aberration ross all model 1.04. Parameter confidence intervals were also generally small, and covariance between parameters was low, supporting that the model could be adequately fit across this diverse dataset.

In the second stage of the fit, parameters relating to cell death pathways were obtained. As with the DNA repair model, data was collected for a range of different cell lines, genetic backgrounds and irradiation conditions, and the model was fit using Scipy’s nonlinear least-squares regression, with the best fitting DNA repair model parameters used as a fixed input. Robust parameters were once again obtained, although survival data is subject to more heterogeneity and significant outliers than the DNA damage data (mean χ^2^=7.7, dominated by a small number of outliers with individual χ^2^>100).

Finally, to enable RBE predictions, *E_DSB_* was fit. A nonlinear least squares regression was carried out, varying *E_DSB_* to maximize correlation between the model’s predicted MID for a given exposure and those experimentally observed in the Paganetti dataset, using Scipy’s nonlinear least-squares regression. The PIDE data was deliberately not used in this fit, but retained as a testing dataset to both confirm the model’s ability to predict RBE in proton data, as well as its ability to extrapolate from a fit performed on protons to other radiation qualities.

### Code Availability

The Medras model has been made publicly available on Github. The analytic version of the model is available at https://github.com/sjmcmahon/MEDRAS, while the Monte Carlo implementation is available at https://github.com/sjmcmahon/Medras-MC. A current version of the code is also available as **Supplementary Material** to this paper, but these models are undergoing continuing evolution and up-to-date versions will be available online.

## Results

### DNA Repair Kinetics

A characterization of Medras’ ability to predict the kinetics of DNA repair is shown in **Figure 2**. Here, model predictions for the kinetics of physical breaks (solid line) and visible foci (dashed line) in repair competent cells are shown, compared to relevant experimental observations. In **Figure 2B**, points show physical breaks measured *via* Pulse Field Gel Electrophoresis (PFGE) or Premature Chromosome Condensation (PCC), while in **Figure 2C** points show the yield of foci. Good agreement is seen with both types of damage, suggesting that this two-stage model with only a simple categorization of simple and complex damage can effectively reproduce results between these different approaches.

These panels currently focus on repair-competent cells for brevity, but the model has also been shown to effectively reproduce repair kinetics in a range of cell lines with DNA repair defects, as presented in previous work (27).

**Figure 2D** shows further validation of this by considering data from ATM-deficient cells which has been plotted for both physical breaks and foci. In ATM-deficient cells, a subpopulation of breaks have long-term repair failure, here modelled as 22% of the total breaks. Both physical breaks and foci show the same impact of this knockout, on both the initial kinetics as well as long-term levels of damage, further supporting the ability of the model to classify damage in this way and effectively reproduce observed repair kinetics.

### DNA Repair Fidelity

**Figure 3:**
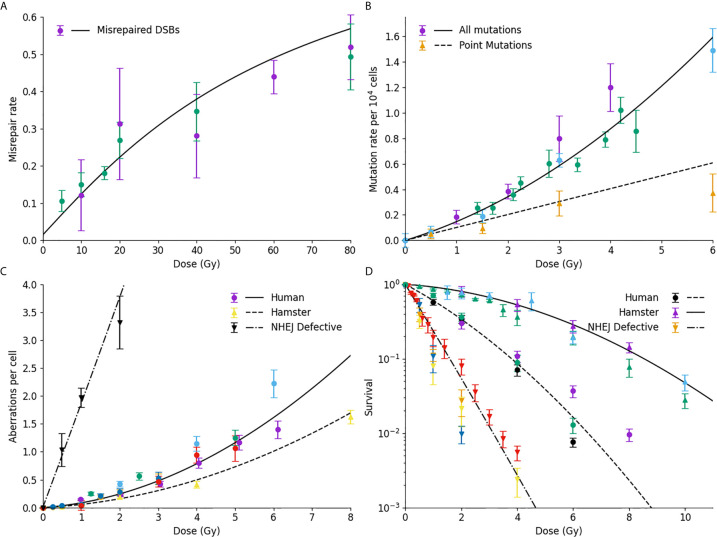
Comparison of model prediction and misrepair endpoints. **(A)** Model prediction (line) compared to observed rates of DSB misrejoining. **(B)** Mutation rates of HPRT gene, considering either all mutations (circles, line) or only point mutations (triangles, dashed line); **(C)** Chromosome aberration yield, for normal human cells (circles, line), human-hamster hybrid cells (upwards triangle, dashed line) or NHEJ-defective human cells (downward triangles, dash-dot line); **(D)** Cell survival for normal Chinese hamster (solid line, triangles), normal human (dashed line, circles) or NHEJ-defective hamster (dash-dot line, downward triangle) cells. For all plots, colours are used to indicate different data sources.

A summary of key Medras predictions relating to DNA repair fidelity and cell survival is presented in **Figure 3**, covering DSB misrepair, mutation yields, chromosome aberrations and cell survival in a selection of systems.

**Figure 3A** shows a comparison of Medras predictions (line) against experimental observations of DNA DSB misrepair measured by PFGE. The updated repair kinetic model effectively reproduces the yield of misrepair over the entire dose range, ranging from 5 to 80 Gy. Similarly, **Figure 3B** shows good agreement between model predicted rates of mutation (solid line) and experimentally observed mutations in the HPRT gene in a variety of studies in hamster lines. Significantly, the model also provides a good estimate of the rate of intra-gene point mutations compared to experimental observations, based on the spatial and genetic distribution of breaks (dashed line).

**Figure 3C** presents data on the yield of chromosome aberrations in a number of systems. Much of the data in this work has been obtained for human lines, and good agreement is seen with model predictions (solid line). However, it can be shown that by taking into account differences in genome size and chromosome number, the model also effectively reproduces the rate of chromosome aberrations in human-hamster hybrid cells (dashed line). Finally, if DNA repair defects are taken into account, the model also effectively reproduces the rate of misrepair in NHEJ-defective cell lines also (dash-dot line). We have also shown that this model provides a good estimate of the fraction of dicentric breaks compared to the total yield (27).

Finally, **Figure 3D** compares observed and predicted survival for a range of cells – Chinese hamster cells (top, solid line), normal human fibroblast (middle, dash line), and NHEJ-defective hamster cells (bottom, dash-dot line). In all three cases, the model effectively reproduces trends in sensitivity across the different lines without any cell-specific fitting, reflecting differences in their underlying genome, DNA repair capability, and cell cycle checkpoints. Of note, for both the normal and repair-defective Chinese hamster cells no direct fitting is performed to the survival data, with survival being entirely predicted from the mechanistic DNA repair model.

### Intrinsic Radiation Sensitivity

As described above, Medras makes no use of cell-specific fitting parameters in its predictions of sensitivity, instead using a simplified phenotypic description to predict cellular responses. Thus, it is possible to compare its predicted radiosensitivity to that observed in a range of cell lines, to evaluate its overall ability to predict intrinsic radiation sensitivity.

**Figure 4:**
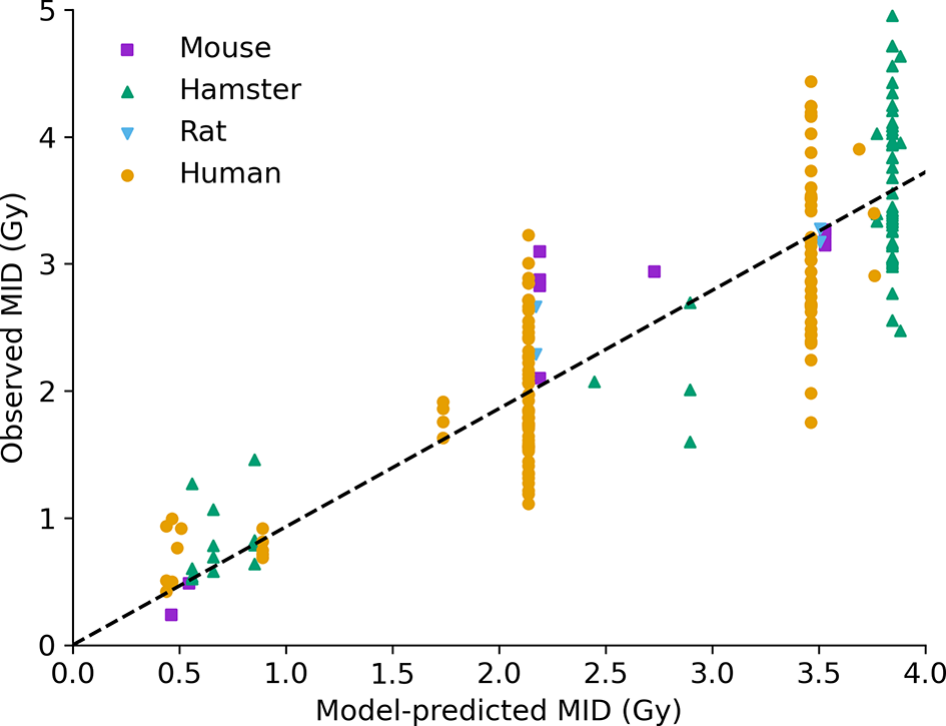
Intrinsic radiosensitivity predictions. Predicted MID for acute X-ray irradiation (x-axis) is compared to observed MID (y-axis) for a range of cell lines (points, coloured by species of origin). The model effectively captures the impact of a range of modifications on radiosensitivity. Best fitting slope line has a slope of 0.93, and an R^2^ of 0.75.

This is illustrated in **Figure 4**, which compares the model-predicted and observed MID for acute X-ray exposure across a panel of more than 200 experimental observations. The majority of these (over 170) are extracted from the PIDE and Paganetti databases, and have not been used to fit any of the DNA repair or cell survival points and thus can be viewed as true predictions.

The performance across the entire range of data is good, with a correlation coefficient of *R^2^*=0.75, and a best fitting slope of 0.93, showing both good correlation and good overall agreement. Good correlation can be seen across a range of cell lines from different species, with different genetic alterations, and different irradiation conditions. Notably, some significant unexplained variance remains among cells with the same model phenotype – seen in the large groups of P53-competent human cells, P53 negative human cells, and hamster cells (around 2.2, 3.4, and 3.8 Gy MID, respectively) showing broad ranges of sensitivity. Possible factors impacting on this will be discussed below, but even taking this into account overall performance is good.

### Impact of High LET Irradiation

**Figure 5:**
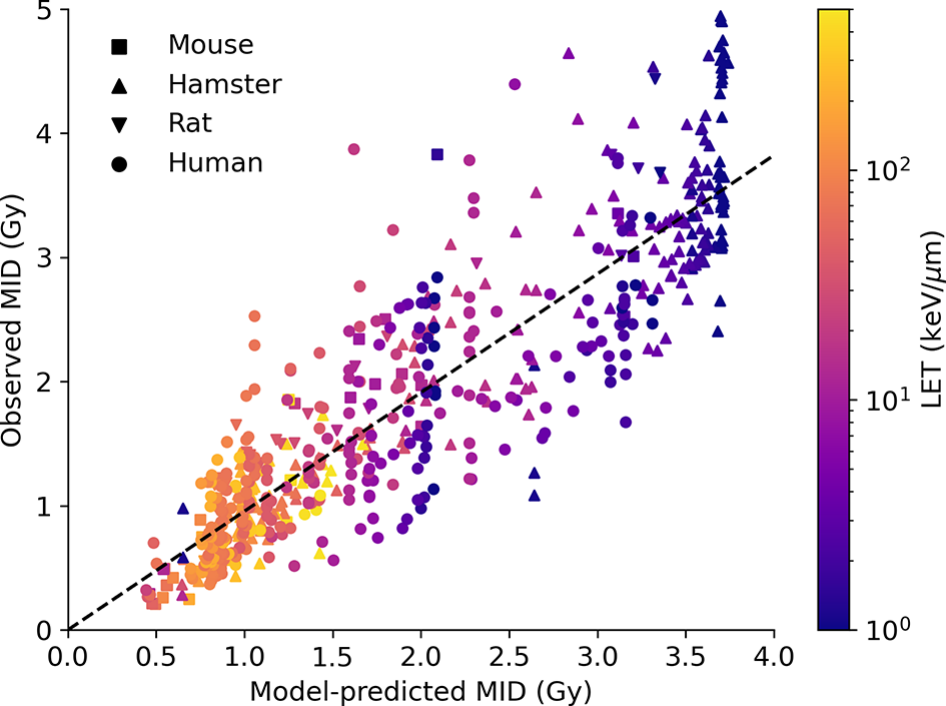
Impact of high LET on radiosensitivity. Predicted MID for acute ion exposures (x-axis) is compared to observed MIDs for a range of cell lines (points, coloured by species of origin) for LETs between 1 and 1,000 keV/μm. Again, the model effectively captures the range of sensitivity, and its dependence on both underlying biology and physics. Best fitting line has a slope of 0.96, with an R^2^ of 0.78.

Similar predictions for a range of different LETs are shown in **Figure 5**. Here, data for both proton and carbon ion irradiations are plotted, compared to experimental observations, for a total of 590 observations, of which 325 are carbon ion exposures and 265 are proton exposures. As with the overall sensitivity prediction above, the overall correlation is good, with *R* ^2^ = 0.78 and a slope coefficient of 0.96. There is also significant heterogeneity, but significantly the model is able to effectively capture the responses across a wide range of LETs and cell backgrounds by fitting a single damage complexity parameter to proton data, and extrapolating this across carbon ion exposures with LETs up to 50 times greater, providing confidence in the underlying mechanistic interpretation. Significantly, this good agreement at very high LETs indicates that the model effectively captures the initial rise and eventual turnover in RBE with increasing LET (being driven by Poisson statistics of arriving tracks) which leads to an increase in MID at very high LETs. Similarly, the model also correctly identifies the negligible impact of elevent LET on RBE, as the death of these cells is dominated by misrepair through the MMEJ pathway, as discussed in previous work (28).

### Impact of Dose Rate

**Figure 6:**
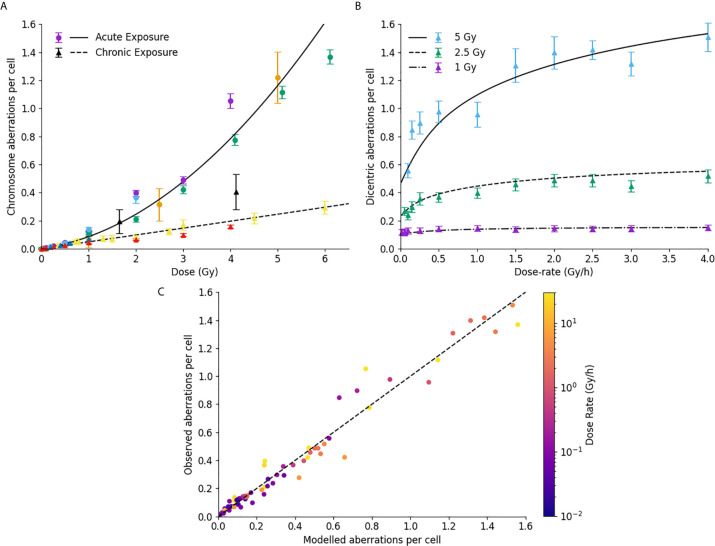
Impact of dose rate on misrepair. **(A)** Comparison of modelled and observed chromosome aberration yields for acute X-ray exposures (circles, solid line) and chronic low dose-rate exposures (triangles, dashed line). Different colours are used to represent different data sources. **(B)** Impact of dose-rate on dicentric aberration yield at a series of different dose levels in human lymphocytes. **(C)** Correlation of modelled and observed chromosome aberration yields, across a range of doses, dose-rates, and underlying biologies, with points coloured according to the delivered dose rate. Best fitting line has a slope of 0.99, with an R^2^ of 0.97.

This paper presented significant improvements in how Medras handles damage which is not induced instantly, enabling it to now incorporate the impacts of dose-rate on a range of endpoints. This is illustrated in **Figure 6** exploring the impact of dose-rate on chromosome aberrations. In **Figure 6A**, yields of chromosome aberrations are compared for human cells irradiated in acute (solid line, >5 Gy/hr dose rate) or chronic (dashed line, <0.1 Gy/hr dose rate) exposures. It can be seen that the updated model effectively distinguishes between these limiting cases, separating out binary misrepair from single-hit misrepair events.

This is further illustrated in **Figure 6B**, which shows the dependence of chromosome aberration as a function of dose, compared to published data for dose rates from 0.05 to 4 Gy/hour. Medras effectively reproduces both the kinetics and magnitude of recovery with low dose rates across several different doses, suggesting it also effectively handles intermediate doses.

To evaluate this over a broad range of conditions, **Figure 6C** presents a correlation plot of modelled and observed chromosome aberration yields for a variety of cell lines, conditions, dose rates and doses. Points have been colored according to the delivered dose-rate. Medras effectively reproduces the observed yield of chromosome aberrations across the whole range considered here, including both low- and high-dose and dose-rate conditions (0.05 to 120 Gy/hour). It also effectively reproduces the observation that DNA repair defective cells are largely insensitive to changes in dose rate (note cell lines with very low dose rates and high yields of aberrations).

For these predictions, it is important to note that only one set of limiting low dose-rate chromosome aberration data was used to fit the underlying model parameters in this dataset (70), with all predictions for intermediate dose-rate recovery emerging from the model kinetic fits to DNA repair.

**Figure 7:**
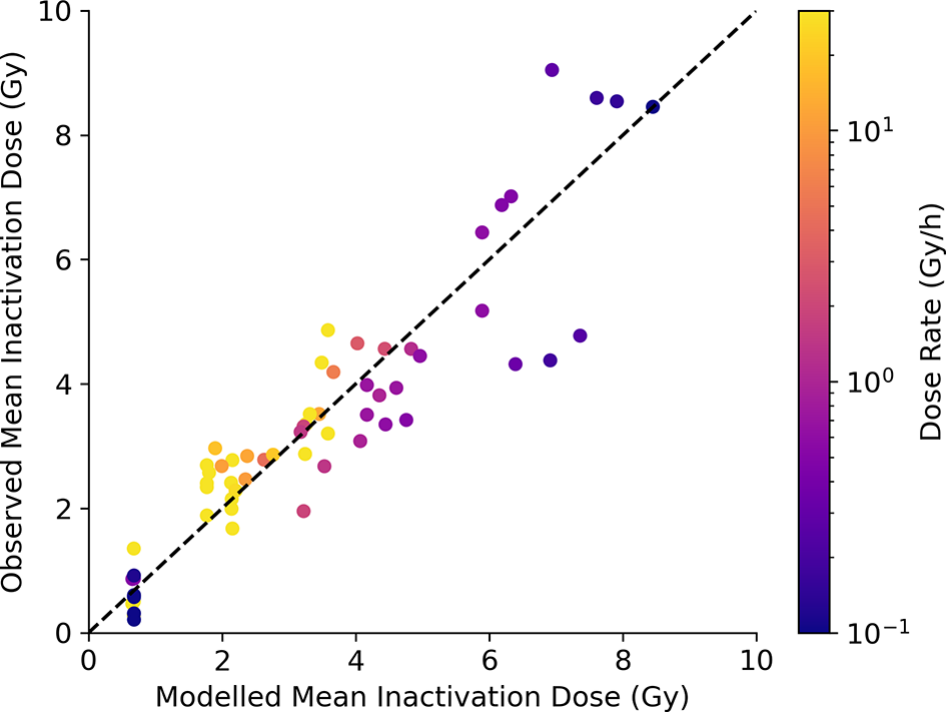
Model predictions of survival at varying dose rate. Modelled MID is compared to observed MID across a range of cell lines and dose rates, with points coloured according to delivered dose rate. Good correlation can be seen, with the increase in radioresistance reflected for cells with competent repair, but not for cells with DNA repair defects (bottom left). Best fitting slope has a slope coefficient of 1.0 ± 0.03, with an R^2^ of 0.84.

Finally, a similar analysis can be performed for predictions of survival. This is shown in **Figure 7**, comparing model-predicted and experimentally observed MID for a selection of exposures at different dose rates. Good correlation is seen across the whole range of sensitivities and dose-rates (an *R^2^* of 0.84 and a slope coefficient of 1.0 ± 0.03), including effectively identifying lines where dose rate is significant and where it is not (E.g. DNA repair defective cells, bottom left). Significantly, this correlation is achieved despite parameters governing the rates of DNA repair being fixed based on fundamental mechanistic mechanisms and not being allowed to vary to improve the quality of the survival fit. Due to limitations in available data a similar MID benchmarking is not possible for fractionation, but illustrations of the ability of Medras to predict the impact of fractionation on dose response is presented in the **Supplementary Information** in **Figure S4**.

## Discussion

Predicting the intrinsic radiosensitivity of cells is of both scientific and clinical interest. After more than a century of research into the radiosensitivity of cells, we now know a great deal about the physical and biological processes which drive cell death and their genetic determinants, but an integrated predictive framework remains elusive, hampering our scientific understanding of this system as a whole. This limitation is a significant challenge to the translation of preclinical knowledge into clinical applications, including the use of intrinsic radiosensitivity as a method for treatment personalization (3, 4).

The Medras model presented here offers a step towards more integrated prediction of radiation sensitivity. This model offers a high-level mechanistic summary of key processes involved in DNA repair, misrepair and cell death, and has been shown to effectively reproduce radiation-induced effects across a range of endpoints including misrepair, mutation, chromosome aberration, and cell death. This integrated approach has a number of advantages over other models which focused more closely on individual pathways or endpoints.

Firstly, by developing a model which mechanistically considers a range of intermediate states before cell death, it is able to naturally generate predictions across a range of measurable endpoints. This means the model is able to be draw on a wide range of types of data to constrain its parameters – spanning over 1,000 measurements of different radiation responses analyzed in this work. Thus, while survival itself depends on more than a dozen parameters, many of these are strongly constrained by other measurements – such as σ on the rate of misrepair as a function of dose – enabling robust, well-constrained fits to be developed. Significantly, this single parameter set also has cross-endpoint predictive power enabling, as in the examples presented here, the impact of dose rate on cell death to be informed by measurements of DNA repair kinetics.

A second key strength of this model is that it involves no empirical cell-specific fitting parameters. While many models require individual fitting parameters as input (such as α and β from the LQ model, or equivalent parameters), predictions in Medras are based on a minimal radiation ‘phenotype’, which contains a small number of explicitly measurable quantities which characterize key aspects of the cell’s radiation response. We have shown that this minimal set of data captures much of the intrinsic sensitivity variation of cell lines, and provides a foundation for more detailed experimental investigations.

These benefits provide a useful complement to much of the radiation response modelling within the literature. A large number of published models have been developed which incorporate predictions of the yield and type of DSBs caused by different qualities of radiation, building on a range of underlying Monte Carlo toolkits to provide models of physical interactions including Geant4-DNA, Topas-nBio, PATRAC and KURBUC (9, 97–102), as well as a number of more empirical and analytic approaches to initial damage and consequent death (103–105). These models provide valuable insights into initial yields and distributions of damage in a range of cell and radiation types. However, in most cases these models apply in ‘generic’ cells, and do not incorporate genetically-dependent features which are known to modulate radiation sensitivity such as DNA repair or activation of apoptosis, and so cannot be used to predict individual sensitivity. There have also been a number of models developed to explore some aspects of biological response, in particular DNA repair pathways, through a range of analytic and stochastic approaches (23–25, 106, 107). These models provide some further insight into the underlying mechanisms of these repair pathways, but are also typically not useful for comparisons between cell lines, as they often involve large numbers of cell-line specific parameters, or do not fully describe the consequences of misrepair and so cannot be linked to biological endpoints such as survival. More detailed discussions on these model differences can be found elsewhere (6). By offering a model which combines sufficient detail in the pathways to reflect the heterogeneity between cell lines with representation of the key biological features of cells, Medras offers a potential way to incorporate knowledge into individual predictions of intrinsic sensitivity.

In addition to its core development, Medras has been used in other mechanistic studies, including investigations of the impact of changing chromosome number and DNA content on radiation sensitivity (108) and the use of different physical and genomic models on the predicted yields of DNA damage and chromosome aberrations (109). It is hoped that by making this code more widely available and providing integration with the SDD format for import of DNA damage data from other models, Medras can help support further investigations in this area.

A number of limitations and challenges do remain, however. One major challenge is that Medras still involves a number of simplifying assumptions about how cells respond to ionizing radiation, including around the nature and spatial distribution of DNA damage, the distribution of DNA within chromosome territories within the nucleus, and the relatively simple binary model of misrepair pathways. All of these can potentially be refined by drawing on additional sources of mechanistic information such as improved Monte Carlo models of DNA damage distributions (109), models incorporating realistic chromosome territories (110), and new systems biology models of the key DNA damage repair and cell death pathways.

The nature and role of damage complexity remains a significant area of potential future development. In the current model, break complexity is treated as a probabilistic binary factor, with breaks deemed as either complex or not, which impacts on the overall repair kinetics and likelihood of repair failure. This repair failure rate is relatively small in repair competent cells the repair failure rate is relatively low and most effects, both at low and high LETs, are dominated by interactions between independent DSBs, rather than local complexity around individual DSBs. However, there is evidence that there may be sub-classes of DSBs which are more difficult to repair due to complexity on a scale of tens to hundreds of bases, due to additional strand breakage, base damage, and other local sequence alterations (111, 112). As this local break complexity depends strongly on LET, this may play a role in the LET-RBE relationship which is currently unaccounted for. Unfortunately, to date there is no clear consensus on what constitutes a complex DSB from the point of repair processes, and thus no robust quantification of these effects which can be used to parameterize models. As a result, Medras’ current model focuses on binary misrepair as a driver of lethality, which has been shown to effectively capture key trends in radiation sensitivity across a wide range of scenarios. Future work drawing on additional data sources, such as precise quantification of DSB complexity or Monte Carlo simulations on the base-pair scale may enable these two contributions to lethality to be separated and understood in more detail.

One other major challenge in this area is the degree of data heterogeneity seen in radiation response data, particularly relating to survival. While many studies of mechanistic endpoints show relatively consistent results (as seen in **Figures 2**, **3**), survival measurements are subject to significant heterogeneity, even for cell lines which are believed to respond similarly (**Figure 4**). However, how much of this variation reflects real underlying biology remains an outstanding question. It is now widely acknowledged that challenges in dosimetry in a range of experimental systems can introduce uncertainties on the order of 20-30% in reported doses and derived sensitivity parameters (113, 114). In addition, extensive sequencing studies have shown significant genetic differences in cell lines once they have been cultured in different laboratories, in many cases dramatically changing their sensitivity to targeted therapies (115). This potential variation is supported by reports of variations of 15-30% in published radiosensitivity parameters across over 100 studies of A549 lung cancer cells, which were not adequately explained by any reported experimental factors (116). A better understanding of these effects, ideally supported by matched characterization and response data, is essential to future model refinement.

If this can be achieved, however, there remains significant potential to deliver novel insights into intrinsic radiation sensitivity and translate this into clinical impact. While the current radiation phenotype parameters in the model depend on direct measurement, many of these parameters are closely linked to particular genetic pathways, which are very well-characterized. If models could be developed which linked these phenotypic parameters to factors which are measurable for patient tumors at the time of treatment – such as gene expression and mutation – then these models could in principle be applied to patient samples as part of the treatment workflow, enabling robust patient sensitivity stratification and the possibility of personalized radiotherapy treatment schedules, incorporating potentially not only overall sensitivity but also variations in, for example, sensitivity to fraction size.

In conclusion, Medras provides a mechanistic model which enables prediction of a range of experimentally and clinically-relevant endpoints, without the use of any cell-specific fitting parameters. This has the potential to be valuable not only for improving our understanding of the processes involved in response to ionizing radiation, but also potential clinical translation of these effects for treatment personalization and optimization. 

## Data Availability Statement

Publicly available datasets were analyzed in this study. This data can be found here: The models developed for this study can be found in Github repositories at https://github.com/sjmcmahon/MEDRAS and https://github.com/sjmcmahon/Medras-MC.

## Author Contributions

SM and KP contributed to the conceptual development and design of the model. SM programmed and developed the model and performed fitting and validation. SM wrote the first draft of the manuscript. All authors contributed to the article and approved the submitted version.

## Funding

This work was supported by a UKRI Future Leaders Fellowship, MR/T021721/1, and a Queen’s University Belfast University Research Fellowship.

## Conflict of Interest

The authors declare that the research was conducted in the absence of any commercial or financial relationships that could be construed as a potential conflict of interest.

## Supplementary Material

Supplementary Data Sheet 1 Supplementary information & results.

Supplementary Data Sheet 2 Medras model code used to generate data in this work.
