# Supplementary material for: A Mechanistic DNA Repair and Survival Model (Medras): Applications to Intrinsic Radiosensitivity, Relative Biological Effectiveness and Dose-Rate
Source: Front Oncol. 2021 Jun 29;11:689112. doi: 10.3389/fonc.2021.689112 (PMC8276175; doi:10.3389/fonc.2021.689112)
Supplement: Supplementary Data Sheet 1 — Supplementary information & results. [file DataSheet_1.pdf]

Supplementary Material

1 Radial energy distributions and misrepair rates

Figure S1 illustrates the steps involved in calculating high-LET effects in Medras. Firstly, radial energy deposition rates are calculated using Geant4 for a range of ions and energies, with protons illustrated below. On the assumption that DSB yields depend only on the total amount of energy deposited in a given volume, these energy distributions can be directly scaled into a DSB distribution by dividing the energy at a given distance by E_{DSB} as described in the main text.

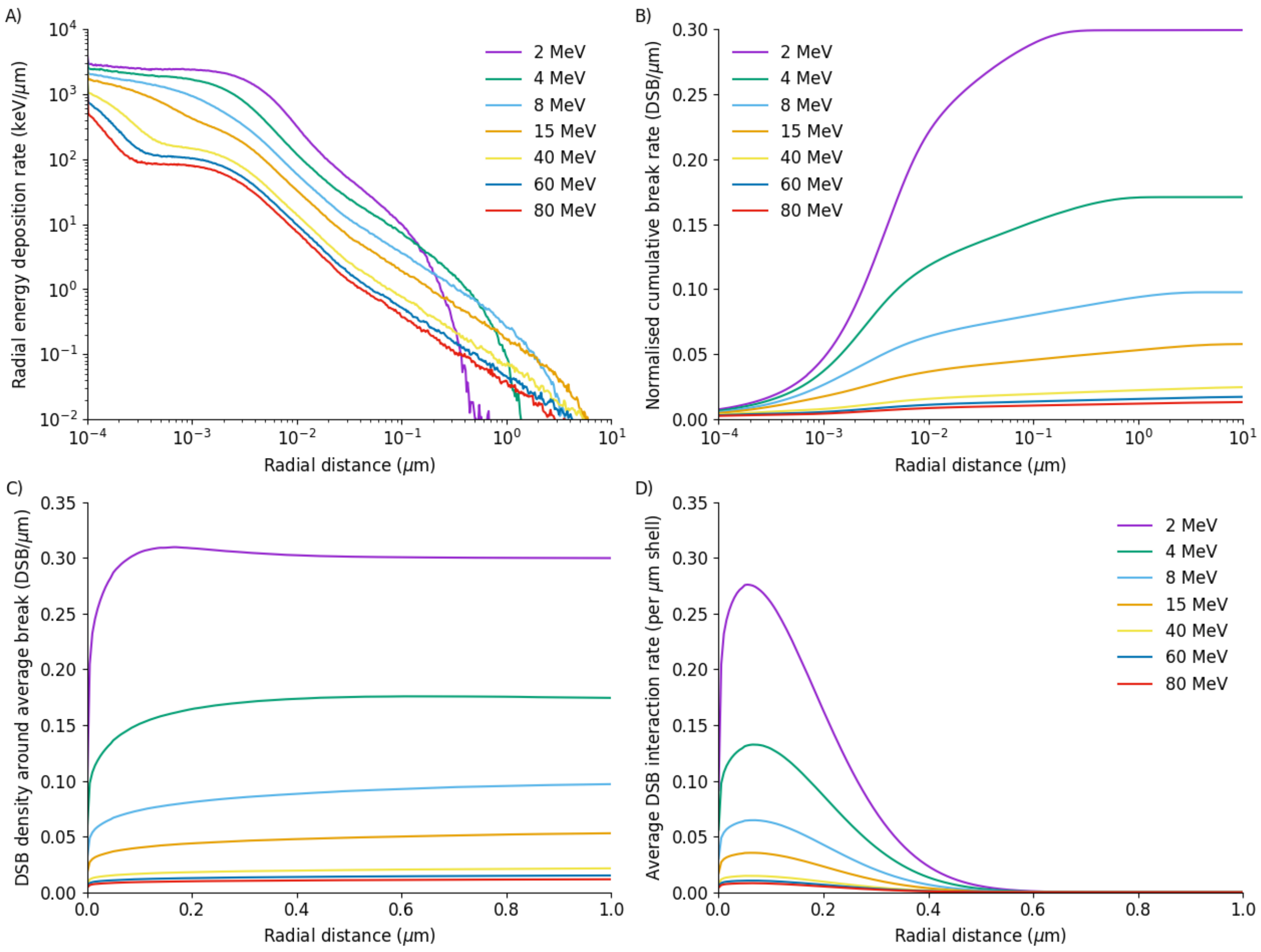

Figure S 1 Illustration of Medras radial track model. A) As described in the main text, radial energy deposition rates were calculated using Geant4 for a range of ions (here showing protons of a range of energies). The energy at each distance was then divided by $\boldsymbol{E_{DSB}}$ to give a rate of DSB induction. B) This can then be used to calculate the cumulative yield of DSB as a function of distance from the track center. C) This radial distribution can then be integrated to give the average number of intra-track DSBs as a function of distance from a randomly selected DSB within the track. D) These DSB densities can then be scaled by the distance-dependent interaction rate described in equation 6 in the main text to give the intra-track interaction rate as a function of distance. The area under this curve then gives the total intra-track interaction rate, which can be added to misrepair predictions as described in the main text.

This can then be used to calculate the expected number of DSBs at a given distance from the track core, the cumulative distribution of which is shown in Figure S1B. This shows that most tracks have a similar structure, with most breaks being created within the first 50-100 nm of the track center, but that the number of DSBs increases significantly as the energy decreases, due to the increase in LET.

This radial distribution can then be integrated to calculate the average separation between a randomly selected pair of breaks within the tracks, as shown in Figure S1C. This has a similar pattern to Figure S1B, and for long ranges is expected to have an equal value, as for radial separations much larger than the track width the shell effectively overlaps small regions of track up- and down-stream of the initial break.

Finally, this break distribution can be scaled by the interaction rate $\zeta = e^{-\frac{d^2}{2\sigma^2}}$, to give the contribution to misrepair at different distances. It can be seen this rises very rapidly as a larger portion of the track is included, peaking around 50-100 nm from the track core, then falling as fewer misrepair events happen at longer distances. The area under this curve then gives the value of the intra-track η component, which can be included in the total response η as described in the main text.

2 Two-stage misrepair solution

As described in the main text, the rate of change in physical breaks can be defined as:

$$\frac{dN_{phys}^{f}}{dt} = -\lambda_f N_{phys}^{f} + p_f k\dot{D}(t) \qquad (1)$$

And that of breaks still bound by repair proteins which would appear in immunofluorescence as:

$$\frac{dN_{prot}^{f}}{dt} = \lambda_f N_{phys}^{f} - \nu_f N_{prot}^{f} \qquad (2)$$

These can be solved numerically for an arbitrary exposure, but for the common case where radiation is delivered in a single acute dose which is short compared to the repair times, a simple analytic solution is available.

Specifically, if an initial number N_0 DSBs are created instantly with no further induction of DSBs, equation 1 can be restated as:

$$\frac{dN_{phys}^{f}}{dt} = -\lambda_f N_{phys}^{f} \qquad (3)$$

With $N_{phys}^{f}(0) = N_f$. This is a standard exponential decay, and has the solution:

$$N_{phys}^{f}(t) = N_f e^{-\lambda_f t} \qquad (4)$$

This expression for N_{phys}^{f} can then be substituted into equation 2 to give:

$$\frac{dN_{prot}^{f}}{dt} = \lambda_f N_f e^{-\lambda_f t} - \nu_f N_{prot}^{f} \qquad 5$$

This is now a standard first-order linear ODE, and assuming $N_{prot}^{f}(0) = 0$, this can be analytically solved to give:

$$N_{prot}^{f}(t) = \frac{N_f \lambda_f \left(e^{-\lambda_f t} - e^{-\nu_f t}\right)}{\nu_f - \lambda_f} \qquad 6$$

Which, if $N_f = N_0 p_f$ is as stated in the main text in equation 4.

3 Repair Pathway choice

As noted in the main text, the pathway through which a break will be repaired is determined based on two factors – firstly, its baseline ‘complexity’, and secondly the availability of the relevant repair pathways. Based on this, breaks will be repaired either through fast, slow, or very slow kinetics. A list of the possible repair pathway choices outlined below. Cells with defects in both HR and NHEJ would be predicted to repair all breaks with very slow kinetics, and cells with defects in both a preferred pathway (HR or NHEJ) and the MMEJ pathway are not considered, due to the lack of data in such cells and the fact that such mutations are typically lethal even in the absence of radiation.

Table S 1 Tabluation of repair pathway choice as a function of cell repair capacity and cell cycle phase. In repair competent cells, ‘simple’ breaks are repaired with fast kinetics and ‘complex’ breaks are repaired by slow kinetics. But if defects exist in these pathways, a portion of the breaks are instead repaired with very slow kinetics via the MMEJ pathway.

**Cell repair capacity**	**Repair Competent**	**NHEJ Defective**	**HR Defective**	
**Phase**	**All**	**All**	**G1**	**G2**
**Probabilities**	$p_f = (1 - p_c)$<br>$p_s = p_c$<br>$p_m = 0$	$p_f = (1 - p_c)(1 - p_{fail})$<br>$p_s = p_c$<br>$p_m = (1 - p_c)p_{fail}$	$p_f = (1 - p_c)$<br>$p_s = p_c$<br>$p_m = 0$	$p_f = (1 - p_c)$<br>$p_s = p_c(1 - p_{fail})$<br>$p_m = p_c p_{fail}$

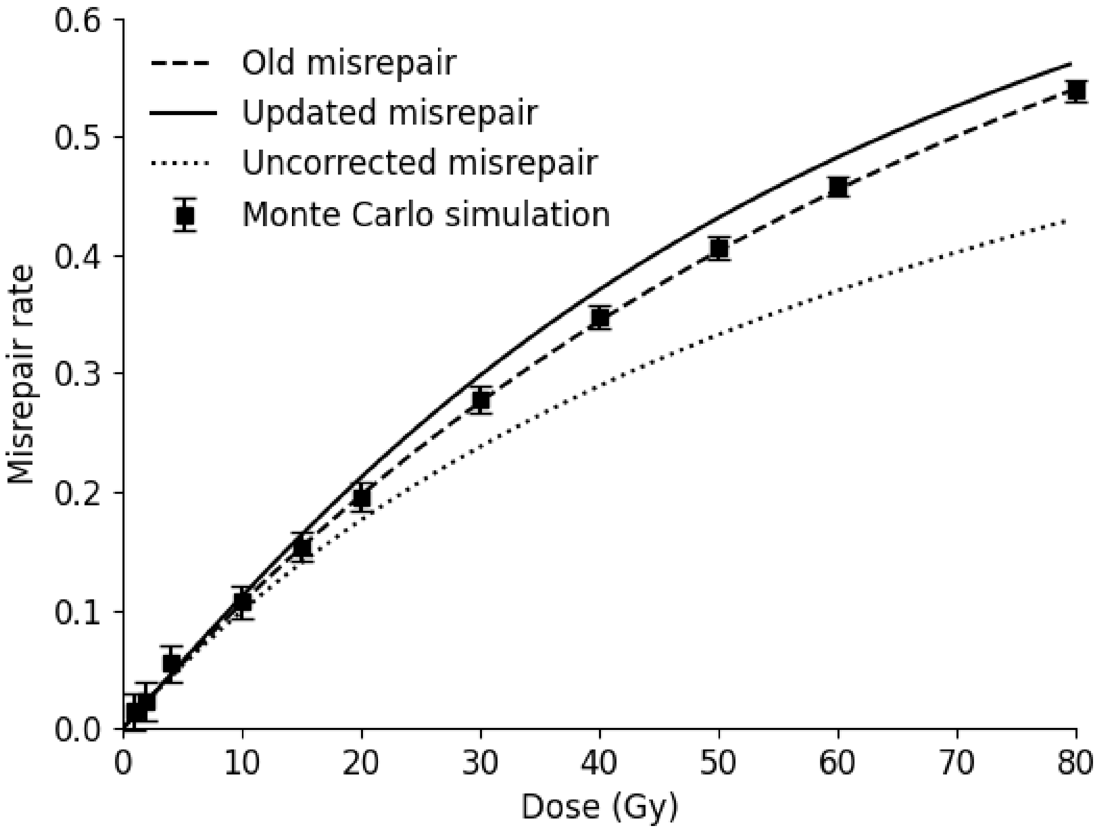

Figure S 2 Comparison of misrepair predictions for different models. The model presented in previous work for acute exposures is shown as the dashed line, showing good agreement with Monte Carlo approaches (points). The modified version used in this work is shown as a solid line, showing agreement to within a few percent at all doses. The dotted line shows the predictions of the simple misrepair model which does not take into account misrepair caused by isolated break ends, which significantly under-estimates total misrepair rates.

4 Comparison of misrepair models

Figure S2 presents a comparison of different models of misrepair for uniformly distributed DSBs delivered in a single dose of e.g. X-rays, for the same misrepair range parameter σ. The dashed line shows the rate used in previous work, for an analytic solution of full repair. It can be seen that this agrees well Monte Carlo predictions as described in the main text, underscoring its accuracy. However, as described in the main text this model cannot be used for fractionated or low dose-rate exposures, as it can only be exactly solved for conditions of full repair.

Instead, in this work an alternative continuous integration approach has been used, which has been shown to agree very well with the previous model across all doses, and in particular clinically relevant doses of less than 10 Gy (solid line). Overall agreement is significantly better than that provided by the uncorrected estimate obtained by directly integrating equation 8 in the main text, which significantly under-estimates the rate of misrepair and overall trend (dotted line).

In practice, this difference leads to a small shift in best-fitting sigma (0.418 in this work compared to 0.428 in previous work), to correct for this error, in which case overall model performance is not significantly changed.

5 Parameter Covariance

Figure S3 shows a heatmap of the uncertainty correlation between different model parameters within the DNA repair fit. Although a large number of parameters are involved in this analysis, it can be seen that many parameters show little or no correlation with one another. This is because, for a majority of these parameters, they are strongly constrained by different classes of experiment, and are thus relatively independent, leading to more stable fits.

Some clusters of correlation are seen – for example in the σ misrepair range parameter and μ repair fidelity parameters, and in the repair rate and foci clearance parameters λ and ν. This typically reflects parameters which act in sequence on a given endpoint and cannot be strongly separated, although overall features which can be directly measured are more stable (e.g. the total repair time for both physical and foci clearance).

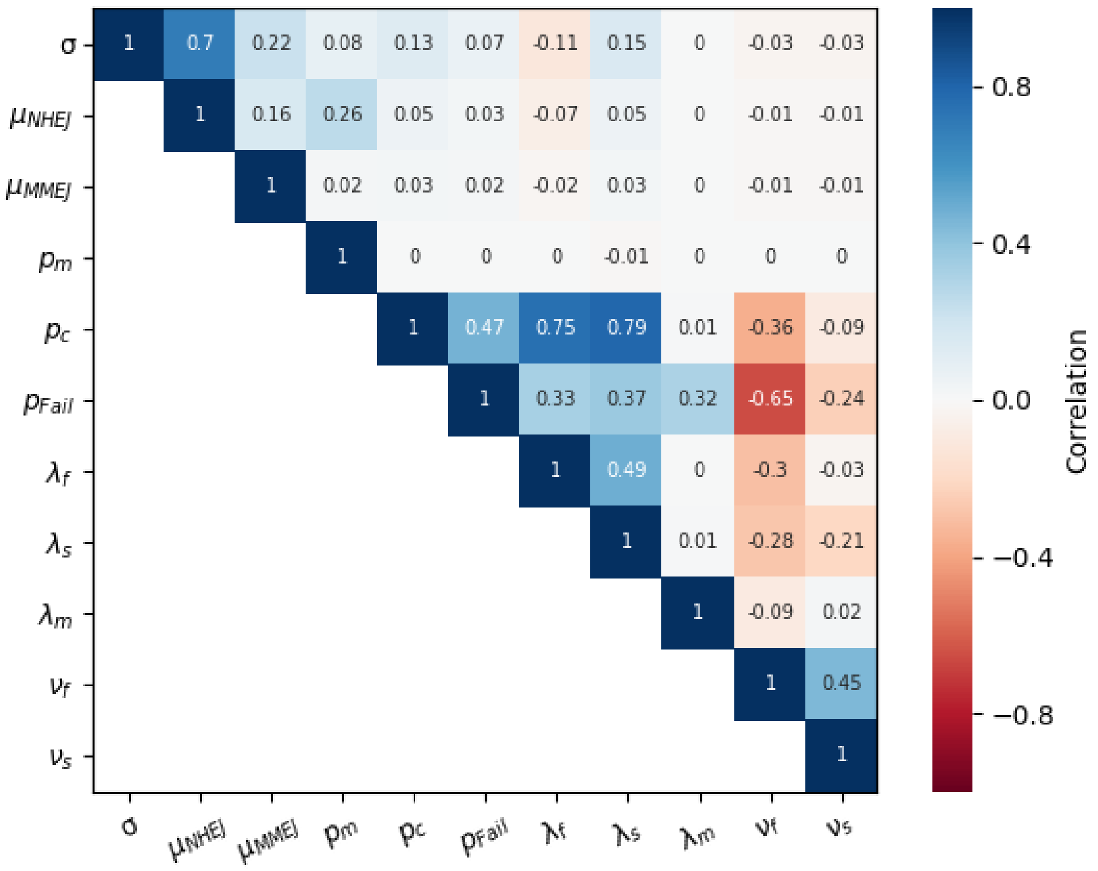

Figure S 3 Heatmap of normalized model correlations for all fitting parameters for DNA repair model. Most parameters are strongly constrained by different classes of experiment, giving limited dependence between misrepair parameters (upper/left) and rate parameters (lower/right).

6 Modelling fractionation effects in Medras

As discussed in the main text, simple fractionation studies can be modelled in Medras by including a period where the dose rate $\dot{D} = 0$ Gy/hr to represent the recovery period between fractions, which can individually be delivered at a high dose rate. Medras effectively tracks the repair of breaks between fractions, and can predict survival for any given pattern of exposure.

This capability is illustrated in Figure S4. This shows the impacts of fractionation as a function of dose (S4A) or time between fractions (S4B). In S4A, dose response curves are shown for a series of doses delivered as either one, two or three fractions with recovery periods of 24 hours, to either normal human cells or NHEJ-defective cells. For normal cells, recovery can clearly be seen, while for NHEJ-defective cells only negligible recovery is seen, as expected. In S4B, the impact of recovery time is shown. Here, doses which give a survival of approximately 1% in an acute exposure (6.6 Gy for repair competent cells, 2 Gy for repair defective cells). The kinetics of repair in the repair competent cells can be clearly seen, corresponding to the repair of DNA damage, while NHEJ cells see little recovery over the same period.

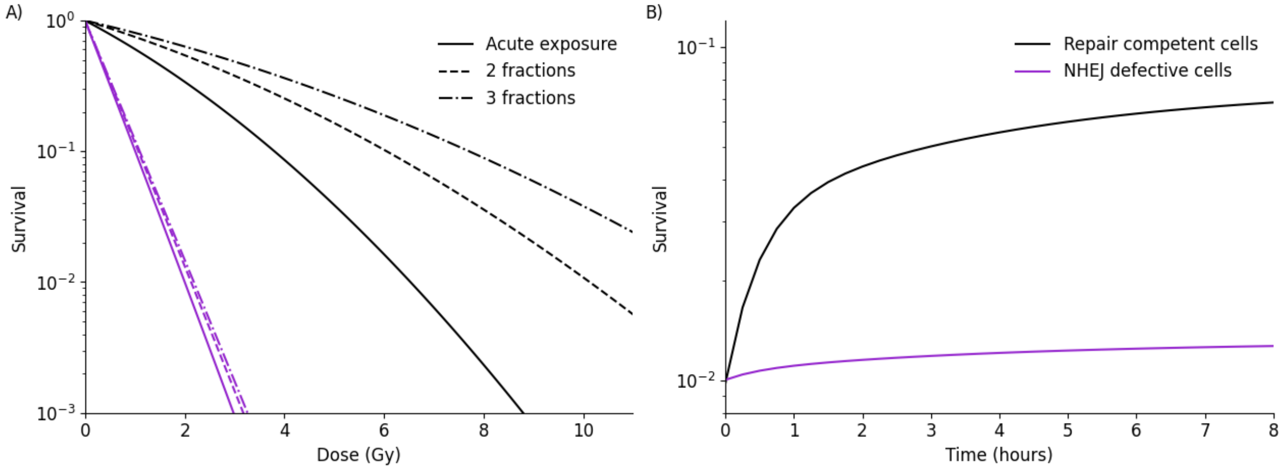

Figure S 4 Examples of fractionated exposures. Panel A shows the response of cells to doses delivered as either 1, 2 or 3 fractions, separated by 24 hours. In repair competent cells (black lines), clear recovery can be seen as fractionation increases, while little benefit is seen in NHEJ defective cells (purple lines). Panel B shows the response of cells to doses of 6.6 Gy (normal) or 2 Gy (NHEJ defective cells) delivered as two equal fractions, separated by varying time. Again, clear recovery is seen in the repair competent cells, but not in the NHEJ defective cells.
